# Clinical efficacy evaluation of combined central and peripheral repetitive transcranial magnetic stimulation for elbow flexor (biceps Brachii) spasticity and upper limb motor function after stroke

**DOI:** 10.3389/fneur.2026.1868183

**Published:** 2026-09-25

**Authors:** Qing Liu, Runhuai Yang, Dongxia Li, Ligang Wei, Erxing Chu, Yangdong Bao, Zhen Liu, Lei Jin, Chunrui Xue, Zhifa Wang

**Affiliations:** 1 Department of Rehabilitation Medicine, The Fourth Affiliated Hospital of Anhui Medical University, Hefei, China; 2 School of Biomedical Engineering, 3D-Printing and Tissue Engineering Center, Anhui Medical University, Hefei, China

**Keywords:** motor function, neuromodulation, rehabilitation, repetitive transcranial magnetic stimulation, spasticity, stroke

## Abstract

**Objective:**

This randomized controlled trial aimed to evaluate the efficacy and temporal dynamics of a combined central and peripheral magnetic stimulation protocol, combining central (M1) and peripheral (Erb’s point) stimulation with conventional rehabilitation, for post-stroke elbow flexor spasticity and motor function.

**Methods:**

One hundred eligible stroke patients with upper limb spasticity were randomly allocated to Group A (*n* = 50) or Group B (*n* = 50). In a 6-week delayed-start design, Group A received active combined central and peripheral magnetic stimulation during weeks 1–3, while Group B received it during weeks 4–6. Both groups received concurrent conventional rehabilitation throughout. Primary outcomes included the Modified Ashworth Scale (MAS) for spasticity and the Fugl-Meyer Assessment for Upper Extremity (FMA-UE) for motor function. Secondary neurophysiological outcomes comprised motor evoked potential (MEP) amplitude and the root mean square (RMS) of biceps brachii electromyography. Assessments were performed at baseline, after 3-weeks and 6 weeks.

**Results:**

A clear phase-specific effect was observed. At 3 weeks, Group A demonstrated significantly greater reduction in MAS scores (2(2, 3) vs. 3(2, 3), *p* < 0.001) and greater improvement in FMA-UE scores (22.2 ± 5.63 vs. 19.2 ± 5.53, *p* = 0.010) compared to Group B. Neurophysiologically, Group A showed an earlier reduction in RMS (20.14 ± 6.71 μV vs. 27.48 ± 7.29 μV, *p* < 0.001) and a transient decrease in contralesional MEP amplitude at 3 weeks. These intergroup differences narrowed at 6 weeks after Group B received the delayed intervention, suggesting that delayed active stimulation may also confer benefit, although spontaneous recovery and concurrent rehabilitation may have contributed to this convergence. Furthermore, the MEP elicitation rate in the affected hemisphere increased more substantially after combined central and peripheral magnetic stimulation treatment, suggesting a significant enhancement of cortical excitability in the affected hemisphere.

**Conclusion:**

A combined central and peripheral rTMS protocol integrated with conventional rehabilitation is effective in alleviating elbow flexor spasticity and improving motor function after stroke. The delayed-start design supports that the observed benefits are attributable to the rTMS intervention, although residual placebo effects cannot be fully excluded. The effects are phase-specific, with motor improvement maintained at 6 weeks and a complex temporal pattern of spasticity reduction, likely mediated by modulating cortical excitability and spinal motor neuron pool activity.

## 1 Introduction

Upper limb spasticity and motor impairment are prevalent and debilitating sequelae of stroke, significantly hindering functional recovery and compromising quality of life (1, 2). Spasticity, characterized by a velocity-dependent increase in muscle tone, arises from an imbalance between excitatory and inhibitory signals within the cortico-reticulospinal pathway, leading to hyperexcitability of the spinal alpha motor neuron pool (3, 4). Traditional management, including physiotherapy and pharmacological agents, often yields suboptimal or transient results, underscoring the need for novel, mechanism-based interventions (5, 6).

Repetitive transcranial magnetic stimulation (rTMS) has emerged as a promising non-invasive tool to modulate cortical excitability (7–10). High-frequency rTMS applied to the ipsilesional primary motor cortex (M1) is believed to counteract post-stroke interhemispheric inhibition, promote cortical plasticity, and subsequently influence spinal cord circuitry (11, 12). While conventional single-target rTMS applied to the motor cortex has shown promise, its effects on spasticity can be inconsistent. A combined central and peripheral magnetic stimulation approach, concurrently addressing central (cortical) and peripheral (spinal) components of the spasticity circuit, may offer synergistic benefits (13–15). Stimulation of peripheral sites like Erb’s point, a superficial brachial plexus site, may directly modulate afferent input and spinal neuron excitability, potentially leading to more robust spasticity reduction (16, 17). By concurrently modulating cortical excitability and spinal-level afferent input, this approach could produce stronger and more sustained therapeutic effects than conventional single-target protocols (18, 19). Preliminary evidence from recent studies has begun to support this hypothesis. Qin et al. (20)demonstrated that combined rTMS and repetitive peripheral magnetic stimulation (rPMS) over Erb’s point significantly reduced spasticity and improved motor function in patients with post-stroke spasticity, while Fawaz et al. (21) reported similar benefits with rPMS in the subacute stage. Krewer et al. (22) also provided evidence for the efficacy of repetitive peripheral magnetic stimulation to increase sensory function in patients with severe limb paresis. Nevertheless, robust clinical evidence for combined protocols remains limited, and the time course and persistence of their benefits are not yet well characterized. An additional key limitation of prevailing sham-controlled trial designs is their inability to isolate the specific contribution of rTMS from the confounding effects of intensive background rehabilitation common to all groups.

To address these gaps, this study employed a randomized, assessor-blinded, delayed-start trial design. The primary objectives were to: (1) evaluate the efficacy of a combined M1 and Erb’s point rTMS protocol on elbow flexor spasticity and motor function; (2) investigate its neurophysiological effects on cortical and spinal/ muscle excitability; and (3) explore the time-course and short-term persistence of the therapeutic effects. We hypothesized that the combined central and peripheral magnetic stimulation would produce superior and phase-specific improvements in clinical and neurophysiological outcomes compared to conventional rehabilitation combined with sham stimulation.

## 2 Materials and methods

### 2.1 General information

This study was a randomized, delayed-start controlled trial. The manuscript was prepared in accordance with the CONSORT 2025 Statement for randomized controlled trials. A total of 100 patients with upper limb spasticity after stroke recovery period from 281 screening potential participants were admitted to the Department of Rehabilitation Medicine (Figure 1), Fourth Affiliated Hospital of Anhui Medical University, from June 2023 to December 2025. These patients had a disease duration of less than 6 months and were aged between 45 and 65 years old. They were randomly divided into two groups using the random number table method, with 50 cases in Group A and 50 cases in Group B. Before treatment, the patient or their family members signed the informed consent form. This research was conducted in accordance with the Declaration of Helsinki of the World Medical Association. This study was reviewed and approved by the Ethics Committee of Chaohu Hospital of Anhui Medical University (Ethics No.: KYXM-202306-007), issued as a single batch approval covering a series of related studies under the same research program, and retrospectively registered in the Chinese Clinical Trial Registry (ChiCTR), registration number: ChiCTR2600130893. And the present participants are entirely independent from those in our previous publication (16), with separate recruitment periods and no participant overlap.

**Figure 1:**
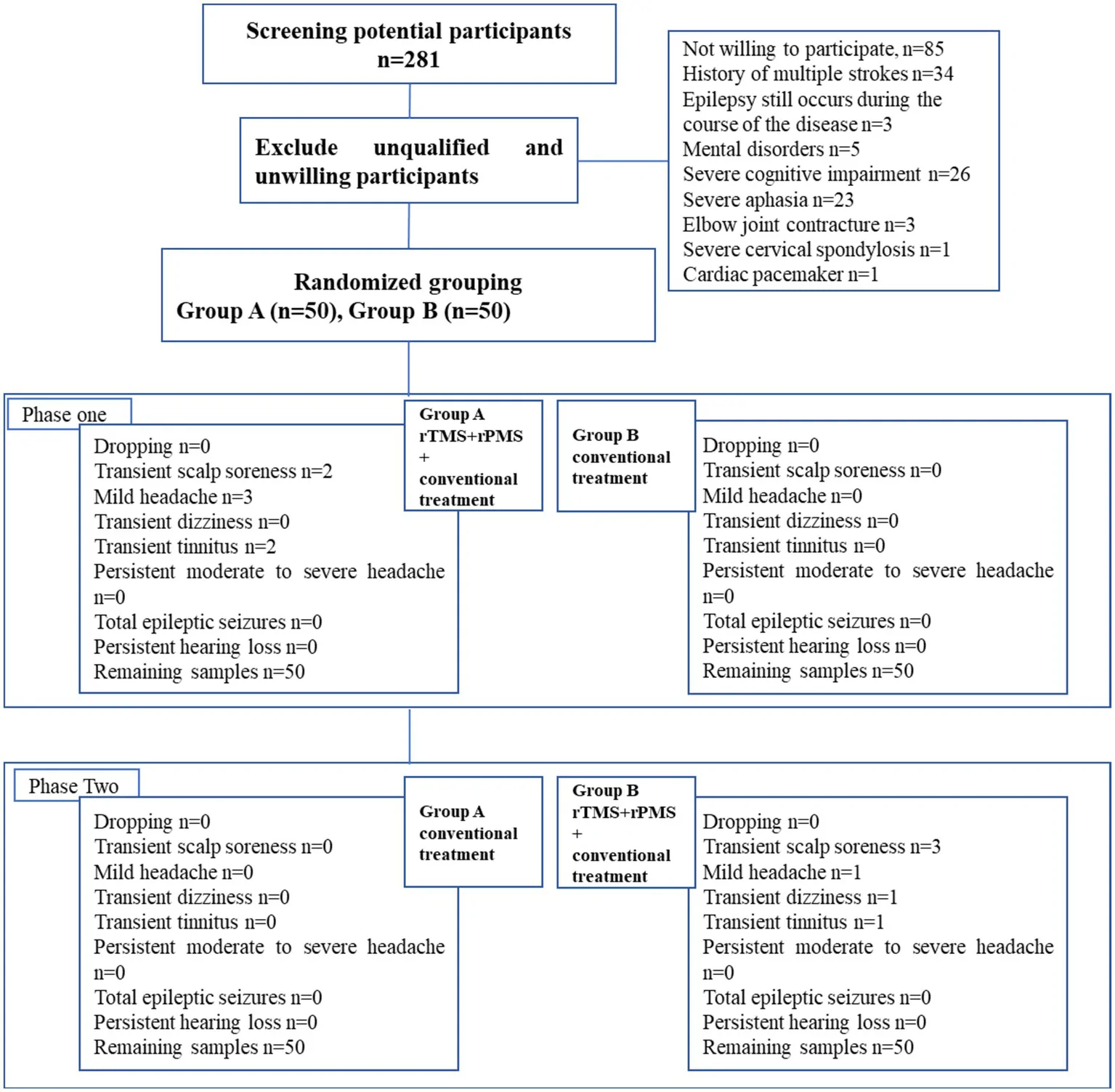
CONSORT flow diagram of the study.

To delineate the independent therapeutic effect of the intervention, a combined central and peripheral magnetic stimulation protocol was implemented, involving simultaneous stimulation of the M1 and Erb’s point. Throughout the 6-week study period, both groups concurrently underwent conventional rehabilitation therapy. Clinical and neurophysiological assessments, including the Modified Ashworth Scale (MAS), Fugl-Meyer Assessment for the Upper Extremity (FMA-UE), motor evoked potential (MEP) amplitude, and root mean square (RMS) of the biceps brachii surface electromyography, were performed at baseline, at the end of the 3-week intervention, and upon completion of the 6-week study period. Although no formal assessment of blinding efficacy was conducted in this study, both participants and assessors blinding were attempted to group allocation throughout the trial. Future studies plan to incorporate post-trial questionnaires or evaluations to examine the integrity of blinding implementation, thereby enhancing the transparency and credibility of the research.

### 2.2 Inclusion and exclusion criteria

#### 2.2.1 Inclusion criteria

All patients participating in this study must meet the following criteria:

1. all patients meet the diagnostic criteria of the National Cerebrovascular Disease Conference in 2019;
2. all patients were confirmed to have stroke through imaging examinations (head CT or MRI);
3. MAS assessment of upper limb spasticity > Grade 1;
4. initial onset of disease leading to limb motor dysfunction;
5. in the recovery phase of stroke, with a duration of less than 6 months.

#### 2.2.2 Exclusion criteria

1. Multiple brain injuries confirmed by head CT or MRI;
2. Oral administration of drugs to reduce muscle tone within 6 months prior to enrollment;
3. Symptoms of joint spasticity;
4. Cardiac pacemaker or deep brain stimulator;
5. Intracranial metal implantation;
6. Pregnancy;
7. History of aneurysm;
8. Uncontrolled epilepsy;
9. Large-area infarction (e.g., infarction area >1/3 of the middle cerebral artery blood supply area), with poor operability;
10. Patients unable to cooperate due to severe cognitive impairment, mental disorders, etc.;
11. Brachial plexus injury;
12. Severe cervical spondylosis of nerve root type.

#### 2.2.3 Blinding

Due to the nature of magnetic stimulation, the therapist who delivered the intervention could not be blinded. However, outcome assessors and data analysts were blinded to group allocation throughout the study. For participants, sham stimulation (coil positioned >5 cm from the skin) was used in the delayed-start group during the first 3 weeks; this positioning produced similar auditory clicking and superficial tactile sensation to active stimulation. However, because active stimulation over Erb’s point may induce noticeable sensory input and muscle contraction, complete participant masking was unlikely despite these efforts. Participants were not informed of their group assignment and were instructed not to discuss treatment sensations with assessors. Blinding efficacy was not formally assessed. Therefore, the potential influence of incomplete participant blinding on subjective clinical outcomes, particularly the MAS and FMA-UE, cannot be fully excluded.

#### 2.2.4 Randomization and allocation concealment

Eligible participants were randomly assigned (1:1) to either the group A or group B. The randomization sequence was generated by an independent statistician using a computer-generated random number table, who was not involved in participant recruitment, intervention delivery, or outcome assessment. Allocation was concealed in sequentially numbered, opaque, sealed envelopes, which were opened only after the participant had completed baseline assessments and provided written informed consent. Enrollment and group assignment were performed by a research coordinator who was unaware of the allocation sequence. Outcome assessors and data analysts were blinded to group allocation throughout the study.

### 2.3 Evaluation method

#### 2.3.1 Assessment of muscle tone

Spasticity assessment: The MAS is used to evaluate the degree of increased muscle tone in patients, primarily assessing the muscle tone of the “flexor elbow” muscle groups (biceps brachii and brachialis) on the hemiplegic side (23). It is divided into six levels: 0, 1, 1^+^, 2, 3, and 4, quantified as scores ranging from 1 to 6 (level 0 = 0 points, level 1 = 1 point, level 1^+^ = 2 points, level 2 = 3 points, level 3 = 4 points, and level 4 = 5 points). A higher score indicates higher muscle tone. The examiner passively moves the joint through its full range, while the patient remains relaxed.

The MAS (Modified Ashworth Scale) muscle tone grading is as follows:

Grade 0: No increase in muscle tone;

Grade 1: Slight increase in muscle tone: When the affected part is passively flexed and extended, minimal resistance is presented or sudden jamming and release occurs at the end of the joint range of motion; Grade 1+: Mild increase in muscle tone: Sudden jamming occurs within the first 50% of the joint range of motion, and minimal resistance is presented during continued movement;

Grade 2: More pronounced increase in muscle tone: When passing through most of the joint range of motion, muscle tone increases significantly, but the affected part can still be moved relatively easily;

Grade 3: Severe increase in muscle tone: Passive movement is difficult;

Grade 4: Rigidity: When the affected part is passively flexed and extended, it presents a rigid state and cannot be moved.

#### 2.3.2 Motor function assessment

The Fugl-Meyer Assessment for Upper Extremity (FMA-UE) is used for motor function assessment, covering shoulder, elbow, and forearm movements (flexion, extension, abduction, internal and external rotation, pronation/supination of the forearm) (20, 24); wrist joint assessment includes flexion, extension, and stability in functional tasks; hand assessment focuses on grasping, pinching, and finger opposition movements; in addition, coordination and speed tests assess repetitive movement ability and movement quality, providing a detailed analysis of motor recovery and functional ability. The FMA-UE scale consists of 33 items, each with 0–2 points (0 point: unable to complete; 1 point: partially completed; 2 points: fully completed), with a total score of 66 points.

#### 2.3.3 Motor evoked potentials (MEP)

MEP is the induced current generated by TMS stimulating the central motor area, which is conducted through the (descending conduction tract) spinal cord and peripheral nerves, and the action potential is recorded on the contralateral target muscle. In this study, the excitability of the unaffected hemisphere cortex was assessed through MEP amplitude. Due to the damage to the corticospinal tract pathway in the affected hemisphere cortex after stroke, MEP amplitude cannot be detected in most patients on the affected side. Furthermore, intervention therapy is performed on the affected upper limb by stimulating Erb’s point with LF-rTMS, which affects the measurement of MEP amplitude in the M1 region of the affected hemisphere cortex through the target muscle of the upper limb. Therefore, this study uses the MEP elicitation rate to assess the excitability of the affected hemisphere cortex.

##### 2.3.3.1 Electrode position

Measurement target: The abductor pollicis brevis muscle is innervated by the primary motor cortex (precentral gyrus) of the contralateral hemisphere. Motor evoked potentials (MEPs) are obtained by repetitive transcranial magnetic stimulation (rTMS) of the M1 region. The recording electrode is placed on the muscle belly of the abductor pollicis brevis muscle of the contralateral upper limb, and the reference electrode is placed on the tendon of the abductor pollicis brevis muscle (at the bony prominence), approximately 3–5 cm away from the recording electrode. The grounding wire is located at the wrist.

##### 2.3.3.2 MEP measurement

The subject is seated comfortably, with the circular coil touching the scalp, and the stimulation point precisely aimed at the target area. The handle is placed at a 45° angle to the midline of the body. For the measurement of MEP on the unaffected side: Set the intensity of repetitive transcranial magnetic stimulation to 120% of the resting motor threshold (RMT), and fine-tune the coil position by manual single-pulse stimulation to find the stimulation hotspot. After the amplitude of the motor evoked potential (MEP) stabilizes, start measuring the MEP amplitude in the primary motor cortex (M1) area on the unaffected side—each measurement includes 15 pulses with a pulse interval of 10 s, and the average amplitude is taken as the result. For the measurement of MEP on the affected side, record the MEP elicitation rate on the affected hemisphere. MEP + indicates that at least 5 out of 10 stimulation trials elicit a motor evoked potential of at least 50uV in the contralateral abductor pollicis brevis muscle at maximum stimulation intensity, with consistent latency. The subject is considered MEP+; whereas MEP− indicates that more than 5 out of 10 stimuli do not elicit a motor potential of around 50uV.

#### 2.3.4 Surface electromyography (sEMG)

This study employs sEMG to assess the degree of upper limb spasticity in patients. Post-stroke upper limb spasticity primarily affects flexor muscles, and muscle tone refers to the contractile force generated during passive muscle activity. By recording and quantifying the excitation level of passive stretching of the biceps brachii through sEMG, the magnitude of muscle contractile force can be determined (25, 26). A higher sEMG value during passive stretching indicates stronger excitability of the muscle during passive stretching, suggesting more severe spasticity. Therefore, observing the changing characteristics of sEMG in the biceps brachii provides a strong basis for the assessment of upper limb spasticity.

##### 2.3.4.1 Electrode position

The patient undergoes sEMG testing in a rehabilitation assessment room with a controlled temperature range of 20 °C to 25 °C. The sEMG acquisition device used is the Dechang Medical SUM-SE-1 sEMG feedback instrument. Two electrodes are placed on the protruding part of the biceps brachii muscle belly, with a center-to-center distance of 3–5 cm between the electrodes, while avoiding the tendon.

##### 2.3.4.2 Measurement method

Fix the affected upper limb and hand on the bracket of the active-passive training device, adjust the sitting position to a suitable distance, and perform continuous mechanical passive movement of the elbow joint on the active-passive training device (Changzhou Siya Medical Equipment Co., Ltd., SYC01-D04). The range of motion for the elbow joint is 0° to 130°, with an angular velocity of 45°/s. Test for 10 s, take 3 segments of baseline stable data, obtain 3 complete original signals, perform root mean square (RMS) of biceps brachii electromyography conversion with a period of 0.1 s, and take the average of the 3 measurements.

### 2.4 Intervention methods

#### 2.4.1 Conventional treatment

Hemiplegic limb functional training (including passive joint mobilization (5 min), therapeutic exercise (30 min), and muscle strength training (5 min)), 40 min per session, once daily, 5 sessions per week, for a total of 30 sessions; Occupational therapy, 30 min per session, once daily, 5 sessions per week, for a total of 30 sessions; Low-frequency pulse electrical stimulation, 20 min per session, once daily, 5 sessions per week, for a total of 30 sessions; Upper limb MOTOmed training, 20 min per session, once daily, 5 sessions per week, for a total of 30 sessions; Paraffin wax therapy, 20 min per session, once daily, 5 sessions per week, for a total of 30 sessions.

#### 2.4.2 rTMS intervention method

The detailed experimental design was illustrated in Figure 2. Both groups of patients received conventional rehabilitation therapy for 6 weeks. Due to the nature of magnetic stimulation, the therapist who delivered the intervention could not be blinded. Outcome assessors and data analysts were blinded to group allocation throughout the study. For participants, participant blinding was attempted using the following sham procedure:

**Figure 2:**
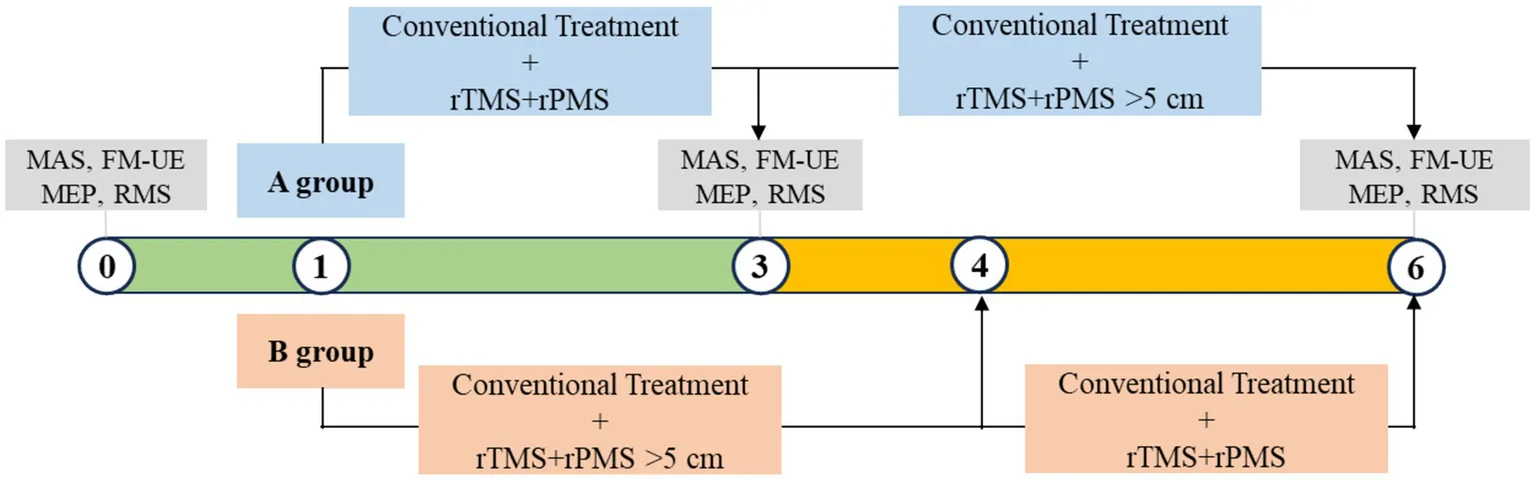
A graphical description of the experimental design.

Patients in Group A received rTMS treatment during the first 3 weeks. Stimulation was first applied to the affected M1 area, immediately followed by stimulation of the affected Erb’s point. Each treatment target was stimulated for 20 min, once daily, 5 sessions per week, for a total of 15 treatment sessions. And at 4–6 weeks, fake stimulation (5 cm away from the skin) was applied to the affected M1 area, immediately followed by stimulation of the affected Erb’s point. Each treatment target was stimulated for 20 min, once daily, 5 sessions per week, for a total of 15 treatment sessions. As noted in Section 2.2.3, complete participant masking was unlikely given the potential sensory and motor effects of Erb’s point stimulation; this limitation should be considered when interpreting the clinical outcomes.

Patients in Group B received fake stimulation (5 cm away from the skin) during the first 3 weeks. Stimulation was first applied to the affected M1 area, immediately followed by stimulation of the affected Erb’s point. Each treatment target was stimulated for 20 min, once daily, 5 sessions per week, for a total of 15 treatment sessions. And at 4–6 weeks, rTMS treatment was applied to the affected M1 area, immediately followed by stimulation of the affected Erb’s point. Each treatment target was stimulated for 20 min, once daily, 5 sessions per week, for a total of 15 treatment sessions.

#### 2.4.3 rTMS treatment protocol

rTMS device model: YRD CCY-I; Manufacturer: Yiruide Medical Equipment New Technology Co., Ltd., Wuhan, China; Coil type: Y125 liquid-nitrogen-cooled circular coil. During stimulation, the patient assumed a comfortable supine or sitting position.

##### 2.4.3.1 Treatment sites

Localization of the affected-side rTMS stimulation area: The C3 and C4 positions were located based on the internationally standardized 10–20 electroencephalogram electrode system. The area 2 cm anterior to these points corresponds to the left and right primary motor cortex (M1) hand area, respectively.

The coil was placed over the M1 hand area projection of the unaffected (contralesional) hemisphere. By gradually adjusting the coil position, the optimal stimulation site that could elicit Motor Evoked Potentials (MEPs) with the shortest latency and largest amplitude was identified. This optimal site on the unaffected hemisphere was then mapped onto the affected (ipsilesional) hemisphere via mirror imaging, and this location was marked as the rTMS treatment target. The M1 stimulation site for each patient was marked daily before treatment to avoid measurement errors and displacement during therapy.

Affected-side Erb’s point: Located 2–3 cm above the midpoint of the clavicle on the affected side, within the interscalene groove between the anterior and middle scalene muscles. The stimulation point was marked daily.

##### 2.4.3.2 Stimulation intensity

Affected-side M1 area: The stimulation intensity for the affected M1 area was set at 120% of the Resting Motor Threshold (RMT) of the unaffected (contralesional) cerebral hemisphere.

Affected-side Erb’s point: The stimulation intensity for the affected Erb’s point was set at 120% of the resting motor threshold of the affected Erb’s point.

RMT measurement: RMT was defined as the minimum stimulation intensity that could elicit MEPs of approximately 50 μV in the contralateral abductor pollicis brevis muscle in at least 5 out of 10 consecutive stimuli.

##### 2.4.3.3 Stimulation parameters

The stimulation parameters were as follows. For central high-frequency repetitive transcranial magnetic stimulation (HF-rTMS) affected-side M1 area, 10 Hz stimulation was delivered in trains of 15 pulses (train duration 1.5 s) with an inter-train interval of 10 s. Each session comprised 104 trains, totaling 1,560 pulses over 20 min. For peripheral low-frequency repetitive peripheral magnetic stimulation (LF-rPMS) affected-side Erb’s point, 1 Hz stimulation was delivered in trains of 12 pulses (train duration 12 s) with an inter-train interval of 2 s. Each session comprised 86 trains, totaling 1,032 pulses over 20 min.

Central 10 Hz rTMS was chosen because high-frequency (≥5 Hz) stimulation increases cortical excitability while concurrently suppressing spinal F-waves, reflecting a dissociation between cortical facilitation and spinal inhibition that is beneficial for spasticity relief (27, 28). Peripheral 1 Hz rPMS applied over Erb’s point activates sensorimotor nerve fibers and mechanoreceptors, enhancing somatosensory and proprioceptive afferents (29). This afferent input reduces contralesional cortical hyperactivation and abnormal transcallosal inhibition, thereby downregulating spinal α/γ motor neuron excitability and decreasing the biceps brachii stretch reflex (30).

Group A received rTMS treatment during the first 3 weeks. Group B received rTMS treatment from week 4 to week 6. Treatment was administered 5 times per week, with each group receiving a total of 15 rTMS sessions.

#### 2.4.4 Measurement of trial data

Before treatment, the MAS and FMA-UE scores of patients in both groups were assessed. MEPs from the M1 area of the unaffected hemisphere and RMS values from surface electromyography (sEMG) of the affected biceps brachii muscle were measured. MAS and FMA-UE scores were reassessed, and MEPs from the unaffected M1 area and sEMG RMS values from the biceps brachii were measured again after 3 weeks and 6 weeks of intervention.

#### 2.4.5 Adverse events

No serious adverse events (e.g., seizure, persistent hearing loss) were observed during the study. Mild adverse events occurred in 13 of 100 participants (13%), including transient scalp soreness (*n* = 5), mild headache (*n* = 4), brief dizziness (*n* = 1), and transient tinnitus (*n* = 3). All symptoms resolved spontaneously without medical intervention. The distribution of mild adverse events was comparable between the two treatment phases: during the first 3 weeks (Group A, active 10 Hz rTMS), 2 cases of scalp soreness, 3 of headache, 0 of dizziness, and 2 of tinnitus were reported; during weeks 4–6 (Group B, active 10 Hz rTMS), 3 cases of scalp soreness, 1 of headache, 1 of dizziness, and 1 of tinnitus were reported. No moderate or severe adverse reactions occurred.

### 2.5 Sample size calculation

The sample size was calculated based on the effect size observed in previous studies of combined magnetic stimulation for post-stroke spasticity. Qin et al. (20) reported a reduction in MAS score from 1.70 ± 0.44 to 1.03 ± 0.61 after combined rTMS and rPMS treatment, corresponding to a large effect size (Cohen’s *d* ≈ 1.26). To be conservative, we assumed a moderate-to-large effect size of *d* = 0.8. With a two-sided *α* = 0.05 and power = 0.80, the required sample size was 26 participants per group (G*Power 3.1). Anticipating a dropout rate of approximately 20%, we aimed to enroll at least 32 participants per group. Ultimately, 50 participants were allocated to each group (total *n* = 100), providing sufficient statistical power to detect clinically meaningful differences. This sample size is also consistent with previous trials: Fawaz et al. (21) used 40 per group for a similar rPMS study, and Krewer et al. (22) included 31–32 per group in a sham-controlled rPMS trial.

### 2.6 Statistical methods

Data analysis was performed using SPSS 21.0 statistical software. Categorical data were analyzed using the chi-square (*χ*^2^) test. For normally distributed continuous data, within-group comparisons were performed using repeated measures analysis of variance (ANOVA). For non-normally distributed continuous data, within-group comparisons were performed using the Wilcoxon signed-rank test, and between-group comparisons were performed using the Mann–Whitney *U* test. The *p*-value < 0.05 was considered statistically significant.

MAS analysis: Because the MAS is an ordinal variable, between-group comparisons were performed using the Mann–Whitney *U* test at each time point, and within-group pairwise comparisons were performed using the Wilcoxon signed-rank test with Bonferroni correction (corrected *α* = 0.0167). Additionally, a responder analysis was conducted, defining clinical response as a reduction of ≥1 grade on the MAS from baseline to each follow-up time point; between-group differences in responder rates were compared using Fisher’s exact test. Results are presented as medians with interquartile ranges and responder proportions with odds ratios where applicable.

Given the delayed-start parallel-group design with repeated measures, two complementary approaches were used: (1) Analysis of covariance (ANCOVA): The week-3 outcome was entered as the dependent variable, group (A vs. B) as the fixed factor, and the corresponding baseline value as the covariate. Adjusted between-group differences with 95% confidence intervals are reported. (2) Linear mixed model (LMM): Fixed effects included group, time (baseline, week 3, week 6), and group × time interaction; participant was entered as a random intercept. An unstructured covariance matrix was specified. The group × time interaction term was used to test whether the trajectory of change differed between groups over the study period. Secondary analyses examined: (a) the between-group difference at week 6 (using ANCOVA with baseline adjustment); (b) within-group changes over time (paired t-tests); and (c) treatment durability in Group A (comparison of week 3 vs. week 6 values) and delayed treatment response in Group B (comparison of week 3 vs. week 6 values). All analyses were two-sided with *α* = 0.05, performed using Python statsmodels.

MEP elicitation rate analysis: MEP elicitation status (elicited vs. not elicited) was assessed at baseline, week 3, and week 6. Because this is a repeated binary outcome, the primary analysis employed a generalized estimating equation (GEE) model with a logit link, binomial distribution, and exchangeable working correlation structure. Fixed effects included group, time (with baseline as reference), and the group × time interaction. Odds ratios with 95% confidence intervals are reported. Simple effects were evaluated using Fisher’s exact test for between-group comparisons at each time point, and McNemar’s test with Bonferroni correction (*α* = 0.0167) for within-group pairwise comparisons. Overall temporal trends within each group were assessed using Cochran’s Q test. A sensitivity analysis using a generalized linear mixed model (GLMM) with random participant intercept was also performed.

## 3 Experimental results

### 3.1 Baseline characteristics

A total of 100 eligible participants were recruited and randomly allocated to either the Group A (*n* = 50) or the Group B (*n* = 50). The flow of participants is detailed in Figure 1. All enrolled participants completed the study protocol, and their data were included in the final analysis. The detailed comparison of baseline characteristics is presented in Table 1. Before treatment, no significant differences were observed in clinical characteristics (age, gender, diagnose, disease duration, MEP, RMS, FMA-UE and MAS) between the two groups (all *p* > 0.05), indicating successful randomization and comparability at the outset of the trial.

**Table 1:** Comparison of baseline characteristics.

Variable	Group A (*n* = 50)	Group B (*n* = 50)	*t*/*Z*/*χ*^2^	*p*
Age	56.3 ± 6.09	57.2 ± 5.14	*t* = 0.834	0.406
Gender (male/female)	32/18	35/15	*χ*^2^ = 0.457	0.499
Diagnose (CI/ICH)	36/14	31/19	*χ*^2^ = 1.131	0.288
Disease duration (*d*)	96.6 ± 28.16	86.9 ± 30.73	*t* = 1.632	0.106
Diabetes	9/41	11/39	*χ*^2^ = 0.250	0.617
*N*_mep+_/*N*_mep-_	5/45	6/44	*χ*^2^ = 0.102	0.749
MEP (μV)	974.60 ± 265.74	935.64 ± 267.55	0.731	0.467
RMS (μV)	29.60 ± 7.60	28.9 ± 10.02	*t* = 0.909	0.366
FMA-UE	14.1 ± 5.05	14.4 ± 5.68	*t* = 0.360	0.720
MAS	2 (2.75, 4)	3(3, 4)	*Z* = −0.066	0.947

CI, cerebral infarction; ICH, intracerebral hemorrhage. *N*_mep+_ denotes the number of MEP+ cases in the affected M1 area, while *N*_mep-_ denotes the number of MEP− cases in the affected M1 area; *χ*^2^ represents the chi-square test value, *t* represents the *t*-test value, and *Z* represents the rank sum test value for ordinal data.

### 3.2 Changes in clinical and neurophysiological outcomes

A clear phase-specific treatment effect was observed across all outcome measures in Table 2, demonstrating the time-dependent impact of the combined central and peripheral magnetic stimulation intervention when integrated with conventional rehabilitation.

**Table 2:** Comparison of before, 3-weeks and 6 weeks treatment.

Variable	Time	Group A (*n* = 50)	Group B (*n* = 50)	*t*/*Z*	*p*
MAS	Before	3(3, 4)	3 (3, 4)	*Z* = −0.066	0.950
	3-weeks	2(2, 3)	3 (2, 3)	*Z* = −5.142	<0.001
	6 weeks	2(2, 3)	2(2, 3)	*Z* = −1.451	0.148
FMA-UE	Before	14.08 ± 5.05	14.44 ± 5.68	0.335	0.738
	3-weeks	22.2 ± 5.63	19.2 ± 5.53	2.643	0.010
	6 weeks	25.32 ± 5.74	24.46 ± 5.85	0.742	0.460
MEP (μV)	Before	974.60 ± 265.74	935.64 ± 267.55	0.731	0.467
	3-weeks	837.80 ± 286.04	976.40 ± 243.50	−2.609	0.011
	6 weeks	873.40 ± 232.98	819.80 ± 241.85	1.129	0.262
RMS (μV)	Before	29.60 ± 7.60	28.96 ± 10.02	0.360	0.720
	3-weeks	20.14 ± 6.71	27.48 ± 7.29	−5.238	<0.001
	6 weeks	23.20 ± 7.92	21.10 ± 7.37	1.373	0.173

#### 3.2.1 Comparison of MAS before and after treatment

As shown in Table 2, No significant between-group difference in MAS scores was observed at baseline (Group A: 3(3, 4) versus Group B: 3(3, 4), *Z* = −0.066, *p* = 0.950). At week 3, Group A showed significantly lower MAS scores than Group B (Group A: 2(2, 3) versus Group B: 3(2, 3), *Z* = −5.142, p = <0.001). At week 6, the between-group difference was not statistically significant (Group A: 2(2, 3) versus Group B: 2(2, 3), *Z* = −1.451, *p* = 0.147). As shown in Table 3, Within-group pairwise comparisons (Wilcoxon signed-rank test with Bonferroni correction, *α* = 0.0167) showed that Group A had significant improvements from baseline to both week 3 and week 6 (both *p* < 0.001), while Group B showed no significant change during the sham period (baseline to week 3, *p* = 0.202) but significant improvement after receiving active stimulation (baseline to week 6, *p* < 0.001; week 3 to week 6, *p* = 0.004). The responder analysis (Table 4) revealed that at week 3, the proportion of patients achieving clinically meaningful spasticity reduction (≥1 grade) was significantly higher in Group A than in Group B (74.0% vs. 42.0%; *p* = 0.002, Fisher’s exact test). At week 6, the responder rate remained higher in Group A (82.0% vs. 58.0%; *p* = 0.016).

**Table 3:** Within-group pairwise comparisons of MAS scores at different time points.

Group	Comparison time points	*p-*value	Corrected significance
Group A	Baseline vs. 3 weeks	<0.001	*
Group A	Baseline vs. 6 weeks	<0.001	*
Group A	3 weeks vs. 6 weeks	0.872	ns
Group B	Baseline vs. 3 weeks	0.202	ns
Group B	Baseline vs. 6 weeks	<0.001	*
Group B	3 weeks vs. 6 weeks	0.004	*

Wilcoxon signed-rank test, Bonferroni-corrected *α* = 0.0167; *p < 0.0167, ns *p* ≥ 0.0167.

**Table 4:** Comparison of clinical responder rates between groups.

Time point	Responder rate (Group A)	Responder rate (Group B)	Fisher’s exact *p-*value	Significance
Week 3	37/50 (74.0%)	21/50 (42.0%)	0.002	**
Week 6	41/50 (82.0%)	29/50 (58.0%)	0.016	*

*p < 0.05, **p < 0.01.

#### 3.2.2 Comparison of FMA-UE before and after treatment

Baseline FMA-UE scores were comparable between groups (Group A: 14.08 ± 5.05 versus Group B: 14.44 ± 5.68, *t* = 0.335, *p* = 0.738). At the 3-week assessment, both groups showed a significant improvement compared to baseline. The improvement in Group A was significantly greater than that in Group B at this time point (Group A: 22.2 ± 5.63 versus Group B: 19.2 ± 5.53, *t* = 2.643, *p* = 0.010). By the 6-week assessment, FMA-UE scores in both groups had further increased and were significantly higher than both baseline and 3-week scores. There was no significant difference between the two groups at 6 weeks (Group A: 25.32 ± 5.74 versus Group B: 24.46 ± 5.85, *t* = 0.742, *p* = 0.460). This pattern is consistent with a phase-specific effect, wherein the integration of rTMS provided a superior boost to motor recovery during its active application period.

#### 3.2.3 Comparison of MEP before and after treatment

Cortical excitability in the unaffected (contralesional) hemisphere, as measured by MEP amplitude, was comparable between groups at baseline (Group A: 974.60 ± 265.74 μV versus Group B: 935.64 ± 267.55 μV, *t* = 0.731, *p* = 0.467). After 3 weeks, Group A exhibited a significant reduction in MEP amplitude compared to its own baseline, and its amplitude was significantly lower than that of Group B at the same time point (Group A: 837.80 ± 286.04 μV versus Group B: 976.40 ± 243.50 μV, *t* = −2.609, *p* = 0.011). Group B showed no significant change from baseline at 3 weeks. At the 6-week assessment, no significant between-group difference was found (Group A: 873.40 ± 232.98 μV versus Group B: 819.80 ± 241.85, *t* = 1.129, *p* = 0.262). Within-group comparisons showed that for Group A, MEP amplitude at 6 weeks was not significantly different from that of 3 weeks. In contrast, for Group B, MEP amplitude at 6 weeks was significantly lower than both its baseline and 3-week values. This pattern is consistent with a transient modulation of contralesional hemisphere excitability during active stimulation; however, the functional significance of this change remains unclear, and it cannot be directly interpreted as evidence of functional recovery.

#### 3.2.4 Comparison of RMS before and after treatment

Baseline RMS values did not differ between groups (Group A: 29.60 ± 7.60 μV versus Group B: 28.96 ± 10.02 μV, *t* = 0.360, *p* = 0.720). At the 3-week assessment, Group A demonstrated a significant reduction in RMS compared to its baseline, with values also being significantly lower than those in Group B (Group A: 20.14 ± 6.71 μV versus Group B: 27.48 ± 7.29 μV, *t* = −5.238, *p* = <0.001). Group B showed no significant change from baseline at this time point. At the 6-week assessment, there was no significant between-group difference (Group A: 23.20 ± 7.92 μV versus Group B: 21.10 ± 7.37 μV, *t* = 1.373, *p* = 0.173). Within-group analysis indicated that RMS values in Group A at 6 weeks were significantly lower than baseline but not significantly different from the 3-week value. For Group B, RMS at 6 weeks was significantly lower than both baseline and the 3-week value. These findings indicate that rTMS treatment led to an earlier reduction in muscle electrical activity during passive movement, suggesting a modulatory effect on spinal cord motor neuron pool excitability that manifests sooner than with conventional rehabilitation combined with sham stimulation.

#### 3.2.5 Comparison of MEP elicitation rate in the affected hemisphere

The changes in motor evoked potential (MEP) elicitation rate in the affected primary motor cortex (M1) before and after the intervention are summarized in Table 3. Following the treatment, the MEP elicitation rate increased in both groups. However, the group receiving conventional rehabilitation combined with sham stimulation showed limited improvement in the MEP elicitation rate on the affected side. In contrast, active high-frequency rTMS combined with conventional rehabilitation led to a more pronounced and early increase in the MEP elicitation rate, suggesting a significant enhancement of cortical excitability in the affected hemisphere. Furthermore, the GEE model demonstrated a significant group × time interaction at week 3 (OR = 3.88, 95% CI: 1.58–9.57, *p* = 0.003) in Table 5, indicating that early intervention increased the odds of MEP elicitation at week 3 by nearly fourfold relative to the delayed-start group. At week 6, the interaction was non-significant (OR = 1.47, 95% CI: 0.48–4.48, *p* = 0.501), suggesting that delayed intervention also improved MEP elicitation, eliminating the between-group difference. Simple effect analyses (Table 6) showed that at baseline, MEP elicitation rates were similar between groups (10.0% vs. 12.0%, *p* = 1.000). At week 3, Group A had a significantly higher rate than Group B (34.0% vs. 14.0%, *p* = 0.034). At week 6, rates were comparable (36.0% vs. 32.0%, *p* = 0.833). Within-group comparisons (Table 7) confirmed that Group A improved significantly from baseline to week 3 (*p* < 0.001) and maintained this improvement at week 6 (*p* = 1.000 vs. week 3). Group B showed no change during the waiting period (baseline vs. week 3, *p* = 1.000) but improved significantly after receiving active stimulation (week 3 vs. week 6, *p* = 0.004). Sensitivity analysis using GLMM yielded consistent results.

**Table 5:** GEE model results for factors influencing MEP elicitation rate.

Variable	Regression coefficient	Standard error	OR	95% CI for OR	*p*-value
Intercept	−1.992	0.435	0.136	0.058–0.320	<0.001
Group (Early vs. Delayed)	−0.205	0.642	0.815	0.232–2.865	0.750
Time (3 weeks vs. Baseline)	0.177	0.176	1.194	0.846–1.684	0.313
Time (6 weeks vs. Baseline)	1.239	0.373	3.451	1.661–7.171	0.001
Group × Time (3 weeks vs. Baseline)	1.357	0.460	3.884	1.577–9.565	0.003
Group × Time (6 weeks vs. Baseline)	0.383	0.570	1.467	0.480–4.483	0.501

*p < 0.05, **p < 0.01, ***p < 0.001, ns *p* ≥ 0.05; Model using exchangeable working correlation structure with robust standard errors.

**Table 6:** Between-group comparison of MEP elicitation rates at each time point.

Time point	Group A	Group B	Fisher’s exact *p*-value	Significance
Baseline	5/50 (10.0%)	6/50 (12.0%)	1.000	ns
3 weeks	17/50 (34.0%)	7/50 (14.0%)	0.034	*
6 weeks	18/50 (36.0%)	16/50 (32.0%)	0.833	ns

*p < 0.05, **p < 0.01, ***p < 0.001, ns *p* ≥ 0.05.

**Table 7:** Pairwise comparisons of MEP elicitation rates within groups at different time points.

Group	Comparison time points	Discordant pairs (Elicited → Not Elicited/Not Elicited → Elicited)	McNemar *p*-value
Group A	Baseline vs. 3 weeks	0/12	<0.001
Group A	Baseline vs. 6 weeks	0/13	<0.001
Group A	3 weeks vs. 6 weeks	0/1	1.000
Group B	Baseline vs. 3 weeks	0/1	1.000
Group B	Baseline vs. 6 weeks	0/10	0.002
Group B	3 weeks vs. 6 weeks	0/9	0.004

McNemar test with Bonferroni correction; after Bonferroni correction, the significance level was set at *α* = 0.0167; *p < 0.0167, ns *p* ≥ 0.0167.

#### 3.2.6 LMM results

The linear mixed-effects model revealed significant treatment effects favoring active stimulation across all primary outcomes (Tables 8, 9). At week 3, after Group A had completed active stimulation and Group B had received conventional rehabilitation combined with sham stimulation, significant between-group differences favoring Group A were observed across all outcomes after baseline adjustment using ANCOVA: MAS (adjusted difference −0.817, 95% CI [−1.12, −0.51], *p* < 0.001), FM-UE (+3.257, 95% CI [1.68, 4.83], *p* < 0.001), MEP amplitude on the unaffected side (−152.005 μV, 95% CI [−252.3, −51.7], *p* = 0.003), and RMS of biceps brachii sEMG (−7.539, 95% CI [−10.42, −4.66], *p* < 0.001). At week 6, after Group B had received the delayed intervention, between-group differences narrowed and became non-significant (MAS: *p* = 0.084; FM-UE: *p* = 0.246; MEP: *p* = 0.273; RMS: *p* = 0.190), indicating that delayed intervention also produced meaningful improvement. Within-group analyses showed that Group A improved significantly from baseline to week 3 (all *p* < 0.01) with no significant deterioration during weeks 3–6, during which they received sham stimulation combined with conventional rehabilitation (all *p* > 0.05), suggesting that the treatment effects were maintained for at least 3 weeks after cessation of active stimulation. Group B showed no significant change during the initial waiting period (baseline to week 3, all *p* > 0.05), but improved significantly after receiving active stimulation from week 3 to week 6 (all *p* < 0.01). The linear mixed model revealed a significant group × time interaction for all outcomes (all *p* < 0.05), indicating divergent trajectories: Group A exhibited rapid improvement followed by a plateau, while Group B showed minimal change initially followed by significant improvement after delayed intervention.

**Table 8:** Comparison of various indicators between the two groups at 3 weeks.

Outcome	Group A	Group B	Coarse difference (A–B)	*T*-test *p*-value	Correction difference	ANCOVA *p*-value
MAS	2.12	2.94	−0.82	0.0000	−0.817	0.0000
FM-UE	22.22	19.24	2.98	0.0096	3.257	0.0001
MEP	837.8	976.4	−138.6	0.0105	−152.005	0.0032
RMS	20.14	27.48	−7.34	0.0000	−7.539	0.0000

*p < 0.05, **p < 0.01, ***p < 0.001; the difference is between Group A and Group B. A decrease in MAS/RMS/MEP indicates improvement, while an increase in FM-UE indicates improvement.

**Table 9:** Inter group differences at 6 weeks (at the end of delayed intervention).

Outcome	Group A	Group B	Coarse difference (A–B)	ANCOVA *p*-value	Significance
MAS	2.1	2.38	−0.28	0.0838	ns
FM-UE	25.32	24.46	0.86	0.2459	ns
MEP	873.4	819.8	53.6	0.2730	ns
RMS	23.2	21.1	2.1	0.1898	ns

*p < 0.05, **p < 0.01, ***p < 0.001; the difference is between Group A and Group B.

## 4 Discussions

This randomized controlled trial investigated the clinical efficacy and neurophysiological mechanisms of a combined central and peripheral magnetic stimulation protocol combined with conventional rehabilitation for post-stroke upper limb spasticity and motor impairment. The principal findings are threefold. First, the integrated intervention demonstrated a clear, phase-specific therapeutic effect, significantly reducing spasticity (MAS) and improving motor function (FMA-UE) during its active application period compared to conventional rehabilitation combined with sham stimulation. Second, neurophysiological assessments revealed that active rTMS induced an early reduction in muscle activity during passive stretch (RMS) and a transient modulation of interhemispheric excitability, as evidenced by MEP amplitude changes in the unaffected hemisphere. Third, the benefits on motor function were maintained at the end of the 6-week observation period, while the anti-spastic effect showed a delayed manifestation in the control group upon later receiving the intervention, suggesting a complex, time-dependent action.

In the present study, the mean age of participants was approximately 56–57 years, which is relatively younger than that reported in many global stroke cohorts. This age distribution was intentional: we recruited patients aged 45–65 years because age is known to influence post-stroke spasticity. Specifically, Opheim et al. (31) demonstrated that older patients have a lower risk of developing long-term upper limb spasticity, whereas middle-aged patients exhibit more severe spasticity in the early phase after stroke. Consequently, our sample may represent a subgroup with higher spasticity severity, which could affect the generalizability of our findings to older stroke populations. Future studies should include a broader age range to validate the applicability of the observed treatment effects across different age groups.

The observed phase-specific superiority of Group A in reducing MAS scores and improving FMA-UE scores at the 3-week assessment strongly supports the additive value of active combined central and peripheral magnetic stimulation to standard care. The subsequent convergence of outcomes between groups at the 6-week endpoint, driven by Group B’s improvement after receiving delayed rTMS, is consistent with a beneficial effect of active stimulation in both groups, although spontaneous recovery, concurrent rehabilitation, and other time-dependent factors may also have contributed. Notably, the mean FMA-UE improvement in Group A at 3 weeks (+8.12 points) exceeded the established minimal clinically important difference (MCID) of 5 points for the upper extremity in chronic stroke populations (32, 33), whereas the improvement in Group B (+4.76 points) fell just below this threshold. This indicates that the early advantage conferred by rTMS was statistically significant and, at the group level, clinically meaningful. By the 6-week assessment, the mean improvements in both groups were well above the MCID (Group A: +11.24 points; Group B: +10.02 points), suggesting that, on average, patients achieved clinically meaningful motor recovery once rTMS was delivered, irrespective of allocation timing. This aligns with previous studies highlighting rTMS’s role in modulating cortical excitability to alleviate spasticity (34–37). However, since the cited MCID was derived from chronic stroke populations and a mean improvement exceeding this threshold does not necessarily imply clinically meaningful change at the individual level, the clinical relevance of these findings in our subacute stroke sample should be interpreted with caution. This “delayed-start” design reduces the influence of confounding by spontaneous recovery and routine rehabilitation, allowing the observed functional gains to be interpreted as consistent with a genuine effect of the rTMS protocol, while acknowledging that residual placebo effects cannot be fully excluded (38, 39). The neurophysiological data offer insights into potential mechanisms. The significant early reduction in RMS values in Group A suggests decreased muscle overactivity during passive movement, which may partly reflect reduced stretch reflex gain. This finding is consistent with a top-down effect in which increased excitability of the affected hemisphere (supported by the rising MEP + rate) enhances descending corticospinal drive, thereby modulating spinal motor neuron excitability. The transient reduction in MEP amplitude in the contralesional hemisphere observed in Group A during active stimulation is consistent with, but not proof of, rebalancing of interhemispheric inhibition. Alternatively, this reduction could reflect non-specific inhibitory effects, changes in attentional state, or measurement variability, and does not directly demonstrate functional recovery. The delayed inhibitory change seen later in Group B may similarly reflect neuromodulatory after-effects of rTMS, although the same alternative explanations apply. Further studies using paired-pulse TMS or other direct measures of interhemispheric inhibition are needed to clarify the underlying mechanisms. It should be noted that both RMS during passive stretch and MEP amplitude are indirect markers of the underlying neural processes. Although MEP amplitude provides information about corticospinal excitability, it may be influenced by multiple factors, including attention, fatigue, and measurement conditions, and therefore does not necessarily reflect functional recovery. Furthermore, the specific contribution of subcortical pathways (e.g., reticulospinal tract) cannot be confirmed based on the current data and warrants direct investigation in future studies.

Moreover, we further conducted a linear mixed model (LMM) analysis to evaluate the longitudinal treatment effects under the delayed-start design. Baseline-adjusted ANCOVA at week 3 showed significant between-group differences favoring early intervention across all outcomes (MAS, FM-UE, MEP amplitude, RMS). Group A showed no significant deterioration through weeks 3–6, while Group B showed significant gains only after delayed stimulation, suggesting that both early and delayed administration may provide benefit within the study period. The LMM revealed a significant group × time interaction, with divergent trajectories aligning precisely with active stimulation periods. These findings support combined stimulation as an effective intervention for upper limb rehabilitation within the 6-week study period, with early initiation accelerating recovery and delayed administration also yielding meaningful benefits.

The study’s key innovation lies in its design and target selection. The sequential intervention schedule not only controls for rehabilitation confounders but also uniquely explores the time-course and short-term persistence of rTMS effects. Furthermore, the combination of a central (M1) and a peripheral (Erb’s point) target represents a novel approach aimed at concurrently addressing both cortical dysregulation and spinal reflex pathways implicated in spasticity. Several limitations must be acknowledged. First, this study was retrospectively registered, with participant enrollment having begun before formal registration, which may introduce potential reporting bias. Second, the sample size, while adequate for detecting the primary effects, may limit subgroup analyses. Third, the lack of a formal assessment of blinding efficacy, though common in device-based trials, is a methodological shortfall. Moreover, because active stimulation over Erb’s point may produce noticeable sensory input and muscle contraction, complete participant blinding was unlikely. Given that the MAS and FMA-UE involve subjective components, the potential influence of incomplete participant blinding on these clinical outcomes cannot be excluded. Future studies should incorporate blinding integrity checks and, if feasible, employ a more convincing sham condition. Fourth, the total observation period was limited to 6 weeks. For Group A, the post-treatment observation period lasted only 3 weeks, and for Group B, week 6 marked the end of active treatment rather than a follow-up phase. Longer-term assessments are therefore needed to determine whether the benefits are durable beyond the study period.

## 5 Conclusion

In conclusion, this study suggests that a combined central and peripheral magnetic stimulation protocol, targeting both the ipsilesional M1 and the peripheral Erb’s point, may provide additional clinical benefit when used as an adjunct to conventional rehabilitation for post-stroke elbow flexor recovery. The intervention was associated with phase-specific improvements in spasticity reduction and motor function during its active application period. The delayed-start design provides some degree of internal control, supporting that the observed improvements are likely attributable to the intervention, although spontaneous recovery and concurrent rehabilitation cannot be fully excluded. Neurophysiological findings offer preliminary evidence consistent with rebalancing of interhemispheric excitability and modulation of spinal motor neuron excitability, though the underlying mechanisms require confirmation through more direct neurophysiological investigations. These results support consideration of this neuromodulation strategy as a potential adjunct in clinical rehabilitation, while recognizing that larger, fully powered, and rigorously controlled trials are needed before definitive clinical recommendations can be made. Future research should prioritize long-term follow-up, optimization of stimulation parameters, and identification of biomarkers for personalized therapy.

## Data Availability

The raw data supporting the conclusions of this article will be made available by the authors, without undue reservation.
